# Precision Medicine in Lymphoma by Innovative Instrumental Platforms

**DOI:** 10.3389/fonc.2019.01417

**Published:** 2019-12-17

**Authors:** Antonello Di Paolo, Elena Arrigoni, Giacomo Luci, Federico Cucchiara, Romano Danesi, Sara Galimberti

**Affiliations:** ^1^Section of Pharmacology, Department of Clinical and Experimental Medicine, University of Pisa, Pisa, Italy; ^2^Unit of Clinical Pharmacology and Pharmacogenetics, Pisa University Hospital, Pisa, Italy; ^3^Section of Hematology, Department of Clinical and Experimental Medicine, University of Pisa, Pisa, Italy; ^4^Unit of Hematology, Pisa University Hospital, Pisa, Italy

**Keywords:** precision medicine, pharmacogenetics, lymphoma, innovative platforms, microarray, GWAS, NGS, ddPCR

## Abstract

In recent years, many efforts have been addressed to the growing field of precision medicine in order to offer individual treatments to every patient on the basis of his/her genetic background. Formerly adopted to achieve new disease classifications as it is still done, innovative platforms, such as microarrays, genome-wide association studies (GWAS), and next generation sequencing (NGS), have made the progress in pharmacogenetics faster and cheaper than previously expected. Several studies in lymphoma patients have demonstrated that these platforms can be used to identify biomarkers predictive of drug efficacy and tolerability, discovering new possible druggable proteins. Indeed, GWAS and NGS allow the investigation of the human genome, finding interesting associations with putative or unexpected targets, which in turns may represent new therapeutic possibilities. Importantly, some objective difficulties have initially hampered the translation of findings in clinical routines, such as the poor quantity/quality of genetic material or the paucity of targets that could be investigated at the same time. At present, some of these technical issues have been partially solved. Furthermore, these analyses are growing in parallel with the development of bioinformatics and its capabilities to manage and analyze big data. Because of pharmacogenetic markers may become important during drug development, regulatory authorities (i.e., EMA, FDA) are preparing *ad hoc* guidelines and recommendations to include the evaluation of genetic markers in clinical trials. Concerns and difficulties for the adoption of genetic testing in routine are still present, as well as affordability, reliability and the poor confidence of some patients for these tests. However, genetic testing based on predictive markers may offers many advantages to caregivers and patients and their introduction in clinical routine is justified.

## Introduction

The innovative platforms for genetic analyses, like genome-wide association studies (GWAS), next-generation sequencing (NGS) or droplet digital PCR (ddPCR), have brought new possibilities for neoplastic disease recognition and classification, as well as for monitoring mutational burden over treatments. By using NGS, for example, it is possible to operate new disease classifications (based on changes in nucleotide sequence and transcriptional levels) and to discover rare albeit important mutations, to investigate the interactions among different pathways that lead to cancer and to analyze epigenetic factors. Moreover, genetic analyses have demonstrated that histopathological lymphoma subtypes are heterogeneous, hence the evaluation of clinical efficacy of new therapies may result in unexpected outcomes for patients (1).

The evolution of bioinformatics has helped this evolution by new instruments and tools capable to manage thousands of information per sample. Noteworthy, these approaches have been flanked by the interrogation of the microenvironment and its different cellular subpopulations, while innovative liquid biopsies for solid and hematological hematological neoplasms (for example, lymphomas) have taken advantage from the high sensitivity of these new platforms.

According to Authors' opinion those new platforms may also be adopted for pharmacogenetic analyses, intended as the study of germinal allelic variants and mRNA levels that may affect drug pharmacokinetics and pharmacodynamics, because the clinical outcome of treatments may depend on several genes, allelic variants or time-changing profiles of transcriptional activities (2).

Below, we discuss the different possibilities offered by these new instrumental platforms, from target discovery up to drug development and, finally, their translation into the clinical practice.

## The “Millennial” Pharmacology and Pharmacogenetics

The way by which we can identify druggable targets, produce effective drugs and optimize treatments based on the patient's genetic profile is dated at the end of the last century. The first breakthrough approach was imatinib for the treatment of patients affected by chronic myeloid leukemia (CML) (3) because the rational development of the drug was guided by the recognition of the BCR-Abl fusion protein as the primary cause of leukemia. After imatinib, several small molecules have filled the armamentarium of oncologists and hematologists, sooner accompanied by monoclonal antibodies directed against (overexpressed or deregulated) extracellular targets that may be responsible for tumor initiation and/or proliferation. The growing knowledge of biochemical pathways involved in cell functions and activities is still playing a significant pivotal role in the search for novel targets (4).

The development of target therapies has been paralleled by a change in the methods and techniques used to investigate genetic variants and gene transcription. We may refer to the “classical” approach as the analysis of candidate genes that are directly involved in drug pharmacodynamics or pharmacokinetics, and the real-time polymerase chain reaction (RT-PCR) is one of the widest used platforms. Its affordability and reliability ensure its wide diffusion among laboratories, and it is the technique of choice to evaluate several candidate genes or variant alleles. Moreover, it could play an important role in the validation process of those markers (i.e., variant alleles or different mRNA levels of target genes) previously discovered by high throughput techniques.

The availability of innovative platforms characterized by maximum sensitivity and high productivity has revolutionized the technical approach by which researchers are evaluating new molecular predictive biomarkers. Microarrays, GWAS, NGS (or massive parallel sequencing) allow the discovery and the analysis of a wide number of pharmacogenetic markers at the same time, overcoming hurdles such as the poor quality of genetic material extracted from formalin-fixed paraffin-embedded (FFPE) sections or the low quantity of DNA and/or RNA harvested from liquid biopsies. Moreover, these platforms can investigate rare or ultra-rare variants. Therefore, in the following paragraphs, the question will be what the innovative platforms are bringing for pharmacogenetics/pharmacogenomics and whether these new approaches are useful for researchers, caregivers and mostly for the patients.

### How Sensitive and Specific the Method Could Be

Pharmacogenetic and pharmacogenomic studies can collect large amounts of genetic material from peripheral blood or tissue sections, so that technique sensitivity (i.e., PCR) is not an issue. Analogously, thanks to primers designed to be employed in allele-specific PCR (AS-PCR) and the amplification refractory mutation system PCR (ARMS-PCR), the specificity is always excellent. For example, a Spanish collaborative group adopted a quantitative RT-PCR (qRT-PCR) analysis to investigate every possible association between progression-free survival (PFS) and the expression of 6 candidate genes in patients affected by B-cell lymphomas (5). Of note, the analyses were performed on mRNA extracted from plasma exosomes, and findings demonstrated that gene expression of *c-myc* and *bcl-6* predicted PFS after first-line chemotherapy (Table 1). The sensitivity of RT-PCR may be increased by adopting a nested procedure as performed by Xu et al. (6). Indeed, a nested AS-PCR was able to identify variants within the *btk* gene at positions c.1634-1635 that were predictive of the poor response to ibrutinib and the early treatment failure with sensitivity equal to 0.8% and with more than 2 cycles of difference from the wild-type allele. Thanks to its sensitivity (10^−4^), AS-PCR is appropriate for specific investigations of candidate genes or variants belonging to genetic signatures, even if the sensitivity of RT-PCR may reach that of droplet-digital PCR (ddPCR) in some cases (14).

**Table 1:** Summary of studies investigating predictive biomarkers in lymphoma patients by less (i.e., qRT-PCR) or more sensitive (i.e., ddPCR and NGS) platforms.

**References**	**Technique**	***N***	**Disease**	**Targets**	**Note**
Provencio et al. (5)	qRT-PCR	98	DLBCL, FL	*bcl-6, bcl-xl, pten, nkkb, akt*	Exosomal gene expression of *c-myc* & *bcl-6* predict PFS after 1st line chemotherapy *c-myc* predicts complete response
Xu et al. (6)	Nested AS-PCR	144	WM	*btk, cxcr4, myd88*	*btk* mutations predicts ibrutinib sensitivity
Xu et al. (7)	AS-PCR	237	WM, MGUS, CLL, MZL, MM, HD	*myd88*	*myd88* mutation as an early oncogenic event in WM pathogenesis Quantitative AS-PCR measures BM involvement
Jimenez et al. (8)	AS-PCR	40	WM, HD	*myd88*	Quantitation of tumor burden in BM Discrimination between mutated and unmutated tissues
Drandi et al. (9)	ddPCR	148	WM, lymphoma, MG	*myd88*	Tumor mutation burden in ctDNA has good correlation with that in BM
Zorofchian et al. (10)	ddPCR	1	Secondary CNS lymphoma	*myd88*	Detection of L256P mutation in CSF
Hiemcke-Jiwa et al. (11)	ddPCR, NGS	23	VRL	*myd88*	Detection of myd88 mutations with high sensitivity and concordance in AH and VF
Hiemcke-Jiwa et al. (12)	ddPCR, NGS	12	LL & PCNSL	*myd88*	100% concordance on tumor FFPE between ddPCR and NGS L265P mutation detected in 75% of CSF samples
Dubois et al. (13)*	NGS	2,015	DLBCL	34 genes (Lymphopanel)	Lymphopanel was informative in 96% of patients Identified molecular heterogeneity among DLBCL subtypes *Tnfaip3* and *gna13* mutations associated with worst prognosis after standard R-CHOP

Additional information regarding gene signatures are presented in Table 4 (*).

*AH, acqueous humor; AS-PCR, allele specific-polymerase chain reaction; BM, bone marrow; CLL, chronic lymphocytic leukemia; CNS, central nervous system; CSF, cerebrospinal fluid; ctDNA, circulating, tumor DNA; ddPCR, digital droplet polymerase chain reaction; DLBCL, diffuse large B cell lymphoma; FFPE, formalin fixed, paraffin embedded; FL, follicular lymphoma; HD, healthy donors; LL, lymphoplasmocytic lymphoma; MG, monoclonal gammopathy; MGUS, monoclonal gammopathy of unknow significance; MM, multiple myeloma; MZL, marginal zone lymphoma; NGS, next generation sequencing; PCNSL, primary central nervous system lymphoma; PFS, progression-free survival; qRT-PCR, quantitative reverse transcriptase-polymerase chain reaction; R-CHOP, rituximab, cyclophosphamide, doxorubicin, vincristine, prednisone; VF, vitreous fluid; VRL, vitreoretinal lymphoma; WM, Waldeström macroglobulinemia*.

When compared to Sanger sequencing or traditional RT-PCR, newer platforms are more sensitive and allow the analysis of large genomic regions or the entire genome. The costs of analysis and instrumentation, together with the need to manage and analyze large amounts of data, represented serious limits to the diffusion of these new platforms, whereas the techniques are now affordable by the majority of researchers.

In the case of germinal variants of genes coding for liver enzymes or transmembrane transporters, the genetic material is always enough to perform the analyses without risking for an early, premature stop. On the contrary, some situations may severely limit the availability of DNA or RNA, as well as in the presence of a small disease burden or for particular sites (i.e., the central nervous system or the eye), which make the biopsy impossible or too risky for the patient. Therefore, the collection of cell-free, circulating tumor DNA (ctDNA) in plasma, the so-called liquid biopsy, represents an optimal and easily accessible source of genetic material, which can be analyzed by techniques with the highest sensitivity (15–17). Moreover, pharmacogenetic analyses can exploit the very limited quantities of nucleic acids released by neoplastic cells in blood, cerebrospinal fluid (CSF) and aqueous humor. The ddPCR meets these expectations by amplifying one single tumor DNA fragment in a volumetrically defined water-in-oil partition, and each final PCR product is analyzed separately from those spotted and amplified on the same support. At difference with ddPCR, the high sensitivity of NGS depends on how many times a DNA sequence is read (the so-called coverage rate): the highest the coverage is (20x or more), the highest is the possibility to identify random mutations. Overall, ddPCR and NGS achieve higher sensitivity (>10^−6^) than quantitative AS-PCR (10^−4^-10^−6^) as demonstrated on the field (18) and, theoretically, they can overpass these limits (19). Thanks to the highest sensitivity, ddPCR and NGS could be useful for the early recognition of new mutated clones within the tumor mass.

Ibrutinib is a specific inhibitor of the Bruton kinase (BTK) and it has been recently approved for the therapy of relapsed/refractory mantle cell lymphomas (MCL), chronic lymphocytic leukemia (CLL) and Waldenstrom macroglobulinemia (WM) (20). The presence of mutations within *myd88* and *cxcr4* genes may severely condition ibrutinib efficacy by triggering several pro-survival signaling pathways (21, 22). A recent study employed nested AS-PCR to identify *btk* gene variants that were subsequently confirmed by NGS (6).

Very recently, ddPCR was applied to detect and monitor *myd88* mutations in peripheral blood. Several studies confirmed that ddPCR was more sensitive (~1.5 log) than quantitative AS-PCR (7, 8) and a high concordance between bone marrow and peripheral blood samples was observed (9). Therefore, the Authors concluded that this technique represents an attractive alternative to bone marrow collection and analysis, especially when unsorted peripheral blood samples with a low burden of tumor cells are available.

The high sensitivity of ddPCR can be helpful when the amount of nucleic acids is very low, as in the case of nucleic acids released in body fluids by neoplastic cells. Indeed, ddPCR was capable to detect *myd88* L265P mutation in 17 vitreoretinal lymphomas (11). In particular, 8 out of 9 patients were positive for the L265P mutation in both vitreous fluid and aqueous humor. Furthermore, the values of sensitivity, positive predictive value and specificity for L265P detection in aqueous humor by ddPCR were 67, 100, and 100% respectively, suggesting that the technique was highly reliable and it may be used as an “*additional minimally invasive tool for accurate diagnosis, detection of recurrence, and monitoring of treatment*” (11).

The ddPCR finds another application in the genetic analyses of the central nervous system (CNS) lymphomas in order to early diagnose and personalize the treatment: the standard procedure of a needle biopsy of the tumor mass may bring severe complications, hence a liquid biopsy in CSF is definitively an attractive alternative (23). Noteworthy, a small volume can be obtained from intraventricular drainage or by lumbar puncture (0.5–5 mL) to prevent possible severe complications associated with the withdrawal of larger volumes. Indeed, the estimated total CSF volume is approximately 100–150 mL in an adult (24). Therefore, the number of cells within the collected CSF could be too low for cytomorphology, cytogenetic evaluations or immunoglobulin gene rearrangement analysis, but they could be sufficient to investigate the mutational burden in target genes. For example, ddPCR has been adopted to investigate the L265P mutations in patients with primary CNS lymphomas (12). After the demonstration that ddPCR and NGS analyses showed a 100% concordance on FFPE sections, Hiemcke-Jiwa et al. demonstrated that ddPCR detected the L265P mutation in 9 out of 12 CSF samples thanks to its high sensitivity. Similarly, ddPCR did enable the identification of the L256P mutation (but not V217F one) in CSF of an 82-years old patient with a secondary CNS lymphoma (10). Therefore, ddPCR represents a reliable technique for pharmacogenetic analyses when high sensitivity and specificity are mandatory in the presence of very limited amounts of DNA.

In addition to these possibilities, the high coverage rate offered by NGS may identify rare or very rare variants. For example, an NGS study recognized molecular subgroups of patients affected by diffuse large B-cell lymphoma (DLBCL) (13). Importantly, in the activated B-cell (ABC) subtype, mutations of *tnfaip3* and *gna13* genes were associated with a poor prognosis after standard chemotherapy (rituximab, cyclophosphamide, doxorubicin and prednisone, R-CHOP regimen).

### Target Abundance

Several allelic variants and/or different gene transcription rates can influence the pharmacokinetics and/or the pharmacodynamics of a specific drug. Therefore, the question is how many targets we must investigate to obtain a good pharmacogenetic signature to minimize the variability.

Microarrays allow the analysis of thousands of genes from different pathways through the evaluation of transcriptional levels, genetic variants (i.e., from genetic variations, polymorphisms, loss of heterozygosity and copy number) and epigenetic features (25). Since the arrays are customizable, the researcher may choose the panel of target genes. Recently, Nanostring technology has expanded these possibilities (26). Both of these methods are currently employed in pharmacogenetic studies to screen for possible variant candidates and then to obtain a signature that may anticipate the effect and the tolerability of chemotherapeutic regimens. Some recent examples are reported below (Table 2).

**Table 2:** Unsupervised evaluation of possible predictive markers in lymphoma patients.

**References**	**Technique**	***N***	**Disease**	**Note**
Martin-Subero et al. (27)	Methylation microarray	367	Hematological neoplasms	220 out of 767 gene hypermethylated, especially *dbc1, dio3, fzd9, hs3st2, mos*, and *myod1*
Wheeler et al. (28)*	GWAS	608	Lymphoblastoid cell lines from different ethnies	Investigated more than 3 million SNPs 41 genes involved in platinum agent cytotoxicity Most of those variants are polymorphic across different world populations
Baecklund et al. (29)	GWAS	586	FL	Investigated ~300.000 SNPs Lymphoma-associated death was strongly correlated with SNPs in 17q24 region near *abca10* and *abca6* genes
Sud et al. (30)	GWAS	22,063	HL	Case-control study Identified 5 risk loci (rs9482849, rs6928977, rs112998813, rs34972832, rs3781093) located within regions of active chromatin with transcription factor binding sites
Sud et al. (31)	GWAS	27,748	HL	Case-control study Identified risk loci for HL inherited susceptibility and dysfunction of immune system
de Jong et al. (32)*	GWAS	1,804	DLBCL	Investigated 3893 genes Identified a 390-gene panel including 9 biochemical pathways Some genes may be targeted by TKI
Mata et al. (33)*	NGS	57	HL	Investigation of mutations in FFPE sections High prevalence of mutations in genes involved in signaling pathways (i.e., GM-CSF/IL-3, CBP/EP300, JAK/STAT, NF-kappaB, B-cell receptor pathways)
Meng et al. (34)*	NGS	6	DLBCL, HD	Evaluation of 2588 miRNA expression 51 differentially expressed miRNA controlled 3631 target genes as well as *tp53* and *fyn*

Additional information regarding gene signatures are presented in Table 4 (*).

*DLBCL, diffuse large B cell lymphoma; FFPE, formalin fixed, paraffin embeded; FL, follicular lymphoma; GWAS, genome-wide association studies; HD, healthy donors; HL, Hodgkin lymphoma; miRNA, micro-interfering RNA; NGS, next generation sequencing; SNP; single nucleotide polymorphism; TKI, tyrosine kinase inhibitor*.

Several mechanisms of resistance contribute to a poor prognosis of tumors. For example, high transcriptional levels of glutathione-S-transferase isoforms were associated with chemoresistance in Hodgkin lymphoma (HL) cell lines (35). Moreover, cancer stem cells (CSC) (36) may be responsible for both reduced sensitivity toward drugs and increased risk of disease progression or recurrence (37). The *in vitro* transcriptional analysis of human B-cell lymphoma cell lines showed that mRNA levels sustained the CSC phenotype in those cells that survived to doxorubicin and phenylbutyrate (38). In particular, among 41 overexpressed genes (such as *jun* and *stat2*), high transcriptional levels of *foxo4* were correlated with high self-renewal capacities, poor PFS and overall survival (OS) in 211 DLBCL patients receiving curative chemotherapy (38).

Although the central role of rituximab in the immunotherapy of follicular lymphoma (FL) (39), physicians should identify patients at high risk of disease progression and address them to alternative therapeutic strategies. Recently, FL patients received rituximab plus different chemotherapeutic regiments, then they were randomized to rituximab maintenance vs. observation for the next 2 years (40). Microarray analysis on fresh-frozen tumor biopsies identified 23 genes associated with individual risk of progression, the signature was confirmed by Nanostring platform in 53 FFPE sections, and subsequently validated in FFPE sections from 488 FL patients enrolled in three distinct studies (40). In conclusion, the signature could be used in FL patients to personalize treatments according to the individual risk score.

The evaluation of epigenetic mechanisms (i.e., methylation, acetylation, transcription control by non-coding RNAs, etc.) likely involved in chemoresistance takes advantages from high throughput techniques. For example, microarray analysis found that epigenetic drugs did modulate gene expression of 233 targets in 11 human lymphoma cell lines of different origin and 480 B-cell lymphoma patient samples with respect to normal B lymphocytes (41). Interestingly, bisulfite sequencing and quantitative methylation-specific PCR (qMSP) identified a 4-gene signature (*dsp, fzd8, kcnh2*, and *ppp1r14a*). The high sensitivity and specificity of the signature were then validated to monitor non-Hodgkin lymphoma (NHL) patients over treatments.

In pediatric patients affected by acute lymphoblastic leukemia (ALL), the analysis of methylated DNA levels was performed by using a microarray chip that included 27,000 CpG sites, while every possible correlation with the resistance to chemotherapy was investigated by unsupervised principal component analysis (42). Results demonstrated that hypermethylation of *adamtsl5* and *cdh11* genes was associated with chemoresistance, as further confirmed by qMSP. Again, the analysis of 770 genes in FFPE sections by Nanostring showed that *ezh2*, a member of the polycomb repressive complex 2 (PRC2) responsible for specific H3 histone methylation at position 27 (H3K27) (43), was highly expressed in extranodal NK/T-cell lymphoma of 10 patients (44). High expression levels of *ezh2* are associated with tumor hallmarks so that the inhibition of EZH2 could be an attractive strategy for lymphoma treatments. Indeed, the study showed that the JAK3 inhibitor tofacitinib can reduce EZH2 expression and H3K27 methylation (44), suggesting that the JAK/STAT3 pathway may control *ezh2* transcription. Therefore, the quantitation of *ezh2* gene expression could help in the optimization of therapeutic plans.

The *hdac6*, a member of the histone deacetylase (HDAC), is involved in the pathogenesis of lymphoma, as demonstrated both *in vitro* (promotion of cell proliferation) and *in vivo* (xenograft growth), it is upregulated in FFPE samples from DLBCL and it decreases the HR23B-mediated degradation of MET (45). Moreover, in an *in vivo* xenograft, the combination of HDAC6 and MET inhibitors (like ricolinostat and crizotinib, respectively) had a potent anti-tumor activity, suggesting its use as “*a promising therapeutic strategy for DLBCL*”.

Very recently, the identification of tumor immune escape caused by CTLA4 and PD1/PDL1-2 pathways (the immune checkpoints) has been an epochal event in oncology and hematology (46). Currently, many monoclonal antibodies directed against these pathways are in clinical development, and those already registered for cancer treatment show impressive activity in several tumors (47–49). The analysis of mechanisms that lead to immune escape, beyond the expression of immune checkpoint markers, could be beneficial to capture new treatment possibilities. In this regard, a meta-analysis performed on 1,446 different transcriptome microarrays from 7 B-cell NHL identified 33 genes (including *ctla4, tigit, pdc1*, and *pdcdlg2*) associated with immune escape (50). That signature was more evident in DLBCL and FL than in MCL and marginal zone lymphoma (MZL). When these data were compared with 5-year overall survival in 580 DLBCL patients, groups III (“*immunogenic tumors with immune escape*”) and IV (“*fully immuno-edited tumors*”) had the worst prognosis as well as group I (“*non-immunogenic tumor*”). Therefore, the Authors proposed that B-cell NHL subgroups with immune escape (i.e., groups III and IV) could find a therapeutic benefit from checkpoint blockade immunotherapies (50).

In another study conducted in 211 DLBCL Chinese patients, Nanostring revealed that the ABC subtype was characterized by high expression of pro-inflammatory genes directly correlated with *pd-l1* and *ido1* expression (51). On the contrary, no associations were observed with several oncogenes like *bad, erbb2*, and *mmp11*. These results suggested that anti-PD-L1 and anti-IDO1 drugs could be more effective in ABC patients.

### Unsupervised Search for Targets

In several cases, predictive markers of treatment efficacy and tolerability are unknown, and the clinical outcome of a chemotherapeutic regimen (i.e., the standard R-CHOP regimen) could depend on variant alleles and/or mutations, intercellular interactions in the extracellular milieu (52, 53) and epigenetic factors (54). For example, histone acetylation, DNA methylation and non-coding RNAs (i.e., long-noncoding or small-interfering RNA) act as modulators of gene expression of transmembrane transporters (55). For these reasons, the investigation of large numbers of possible targets needs appropriate platforms (Table 3).

**Table 3:** Studies focused on the search for putative predictors of toxicity in ALL pediatric patients.

**References**	**Technique**	***N***	**Disease**	**Note**
Stocco et al. (56)*	GWAS	208	ALL children	38 out of 15,661 genes correlated with TPMT activity, especially *pacsin2*
Fernandez et al. (57)	GWAS	3,308	ALL children	*nfatc2* SNP rs6021191 highly associated with HR
Ramsey et al. (58)	GWAS	498	ALL children	Two SNPs in *f2rl1* gene predict pleiotropic effects of dexamethasone (including osteonecrosis and thrombosis)
Kutszegi et al. (59)	NGS	359	ALL children	HLA haplotype (HLA-DRB1*07:01/HLA-DQB1*02:01/HLA-DQB1*02:02) predict HR

Additional information regarding gene signatures are presented in Table 4 (*).

*ALL, acute lymphoblastic leukemia; GWAS, genome-wide association studies; HR, hypersensitivity reactions; NGS, next generation sequencing; SNP, single nucleotide polymorphism; TPMT, thiopurine methyltransferase*.

GWAS was adopted to investigate the correlation among complex traits in the population (i.e., polygenic diseases) and genetic variants because the analysis shows the chromosomal region where the causative gene is (60). Furthermore, findings of several GWAS studies may be combined in meta-analyses to improve power and sensibility of the study. For example, the meta-analysis of 7 GWAS studies, for a total of 5,325 HL patients and 22,423 controls (31), demonstrated that 5 new loci were significantly associated with inherited susceptibility to HL and dysfunction of the immune system, independently from patients' age or histological subtype. A similar study based on three GWAS trials identified a set of loci (with a special representation of transcription factors) significantly correlated with the risk of developing classical HL (30).

In other cases, GWASs investigate possible predictive markers of drug sensitivity or tolerability to optimize pharmacological treatments in the clinical routine. An interesting GWAS in 608 lymphoblastic cell lines reports the identification of several variants associated with cellular response to platinating agents (28). The final signature includes genes correlated with cisplatin and carboplatin sensitivity, such as those involved in the nucleotide excision repair system (*ercc2, ercc6*) and antiapoptotic phenomena (*bcl2*). Furthermore, the Jak1-STAT3 pathway plays a central role in ABC DLBCL, because its sustained activation may promote cell proliferation and survival, and seems a possible cause of chemoresistance, as well as the NFkB pathway (61). On the basis of these observations, the role of STAT3 was further investigated in two human cell lines of ABC DLBCL by GWAS (62). Researchers found that STAT3 directly targets more than 2,000 genes, including those involved in several functions of B cells, as well as activation, migration, survival and proliferation. The further transcriptomic analysis suggested a possible combination between the JAK inhibitor ruxolitinib with lenalidomide to obtain maximum therapeutic benefits, as successfully demonstrated in a xenotransplanted murine model. Finally, the meta-analysis of two GWASs found 4 single nucleotide polymorphisms (SNPs) associated with specific study endpoints (i.e., lymphoma-specific death or progression) in 586 FL patients (29). Among these 4 SNPs, two of them were located nearby *abca10* and *abca6* genes, which belong to the ATP-binding cassette transmembrane transporter superfamily.

An interesting approach employing GWAS has been recently published by de Jong and coworkers (32). Using the Genome Expression Omnibus (GEO) database, the Authors identified a large set of genes known to be correlated with DLBCL pathobiology in 1,804 patients. The comparison of those data with the rituximab target CD20 (in a *guilty-by-association* analysis) led to the discovery of “new” genes (*wee* and *parp1*) that could play a role in DLBCL treatment. Indeed, *in vitro* experiments showed that rituximab plus WEE and PARP1 inhibitors had an increased cytotoxic effect in human cell lines characterized by a poor sensitivity toward rituximab alone. Therefore, GWAS findings allow the selection of new combined treatments, even outside the standard chemotherapeutic regimens.

NGS offers the possibility of sequencing the whole genome (whole genome sequencing, WGS), the exome (whole exome sequencing, WES), the transcriptome or a set of genes of interest, but these options are not equivalent (25, 63), because some of them cannot investigate specific targets (i.e., WGS for GC-rich domains, WES for insertion and deletion). Although these differences, the highest coverage rates (i.e., 100x or more) can identify the rarest mutations. A clear example of NGS application in clinical trials was represented by the analysis of 208 genes in 60,706 unrelated individuals to search for rare variants that could affect drug efficacy and tolerability (64). The study collected results from other NGS trials, and final findings revealed that those rare variants were (a) “stron*gly enriched in mutations*” and (b) they “*account for the entire genetic variability*” in more than 50% of genes. Therefore, the Authors concluded that those rare variants may explain a significant percentage of the interindividual variability in drug metabolism. Another NGS analysis performed on FFPE tumor samples obtained from 57 classical HL patients showed a wide genomic heterogeneity (33). More interestingly, a high mutational burden was detected in genes involved in signaling pathways, such as *bp*/*ep300, jack*/*stat, nfkb*, and *btk*. Some of these genes code for druggable proteins that could be novel promising targets in classical HL.

The evaluation of epigenetic factors that may influence drug efficacy and/or tolerability can be performed by several methods, which may ensure high-throughput with low error rates. Methylation microarrays allowed the investigation of CpG islands and the subsequent identification of hypermethylated genes by using the classical bisulfite conversion. For example, various types of lymphomas had different levels of methylated genes, so that “*DNA methylation profiling could be then a useful approach… to stratify patients for treatment with demethylating agents*” (27). Another study in FL demonstrated that levels of methylated DNA were significantly different in tumor samples and healthy cells (65).

Methylation microarrays are highly standardized and cover a high percentage of CpG, but not all sites. On the contrary, the investigation of genetic methylation profiles in the whole genome or in target sequences may improve when NGS and WGS are coupled with bisulfite conversion, endonuclease digestion or affinity enrichment of DNA (66–69). For example, whole genome bisulfite sequencing (WGBS) can detect a single base regardless of the region where the methylation can occur. The enrichment-based techniques, MeDIP-seq and Methyl Cop-Seq that use antibodies and proteins, enrich genomic DNA before the analysis, even if this characteristic can bias sequence selection toward hypermethylated regions. Finally, findings of reduced representation bisulfite sequencing (RRBS), another enrichment-based method, depend on whether the interested region has restriction sites or not.

Several proteins that control genomic transcription through DNA methylation and histone modifications represent further epigenetic targets that can be assayed by NGS. DLBCL lymphoma subtypes, as well as germinal center B-cell (GCB), primary mediastinal B-cell and ABC lymphomas, showed different percentages of mutations in *ezh2, kmt2d, ep300, mef2b*, and *crebbp* genes (13). In particular, the evaluation of *ezh2* expression and/or mutational status in several hematological malignancies may predict the efficacy of EZH2 inhibitors under development, as well as tazemetostat (70).

Another attractive area to study post-transcriptional regulation of gene expression includes micro-RNA molecules (miRNA), which represent an option to obtain information regarding pathobiology of diseases and their sensitivity toward chemotherapy. In DLBCL, various miRNAs seem to be associated with lymphoma pathogenesis (34, 71). For example, among 2,588 miRNA identified by NGS in serum samples, researchers obtained a list of 51 miRNA differentially expressed (the majority of them were down-regulated) that were involved in the control of over 3,600 target proteins such as TP53 (34). A similar approach performed *in vitro* analyzed miRNA levels in parental and chemoresistant human DLBCL cell lines (72). In particular, 2 out of 37 upregulated targets (miR-99a-5p and miR-125b-5p) were further validated in plasma-derived exosomes from DLBCL patients and their transcriptional levels were correlated with a shorter PFS. The interest for circulating miRNA is further sustained by their role as predictive markers of treatment efficacy, as it occurred for miR-222, miR-181a, miR-18a (71), miR-125b, miR-130a (73), and miR-21 (74).

### Drug Tolerability and Treatment-Induced Toxicities as Targets for New Technologies

Drug-induced toxicities represent serious concerns for patients (i.e., delayed or discontinued therapies, reduced clinical benefits, a worsened quality of life), caregivers (i.e., need for supportive care or drugs, increased workload), and finally for health care systems (i.e., increased overall costs). Since the beginning of pharmacogenetic studies, mRNA levels, polymorphisms or mutations of candidate genes have been explored as possible predictive markers. However, as it happens for clinical efficacy, part of the variable individual tolerability remains unexplained. Therefore, the adoption of new platforms may improve the understanding of adverse drug reactions (ADRs) (75), especially if they are polygenic traits.

An interesting example refers to polymorphisms of thiopurine-methyl-transferase (TPMT) that are often predictive of mercaptopurine-associated toxicities. Stocco et al. performed a genome-wide analysis for both gene expression and SNPs in a panel of human cell lines (56), finding that mRNA levels and polymorphisms of *pacsin2* displayed the best association with TPMT activity (Table 4). Lastly, this relationship between *pacsin2* SNP rs2413739 and TPMT activity was confirmed in ALL pediatric patients, including children who suffered from gastrointestinal toxicity.

**Table 4:** Gene signatures with their genes and polymorphisms as presented in Tables 1–3.

**References**	**Disease**	**Targets (genes or polymorphisms)**
Stocco et al. (56)	ALL	MTMR4, FMNL3, GPC5, CCDC6, MAP3K12, CLEC4A, IL2RA, CETP, SPINT2, C7orf43, PTK2, DLGAP4, NADSYN1, SOCS2, PACSIN2, MAPK11, TGM5, SV2B, DSC2, CMKLR1, CHFR, BASP1, RAB6IP1, FFAR2, C4orf34, CDK2AP1, RAB4B, ETV6, MAP3K8, C1orf34, DSC3, CEP68, RSPRY1, SPRED2, C17orf66, BCL2L11, SSH1, CCND1
Wheeler et al. (28)^a^	*In vitro*^b^	A2BP1 (rs8051396), CELSR1 (rs7293002), CHN2 (rs6968010), DTNB (rs7605235), EIF2S1 (rs8008724), FAM71D (rs10431718), IL28RA (rs3893319, rs6698365), MPP5 (rs10138824, rs2146229), PCDH9 (rs2875481), PDE3A (rs4326884), SEZ6 (rs2277664), other SNPs (rs582894, rs1963399, rs6533942, rs6812672, rs6817737, rs6846333, rs7668874, rs7686539, rs7758889, rs9263567, rs9287508, rs9993212, rs9995393, rs10005313, rs10020267, rs10020294, rs10496537, rs11098326
Dubois et al. (13)	DLBCL	STAT6, XPO1, SOCS1, BCL2, CIITA, TNFAIP3, CD79B, PIM1, GNA13, CD58, CREBBP, B2M, EZH2, TNFRSF14, MFHAS1, MYD88, ITPKB, PRDM1, NOTCH2, IRF4, MEF2B, BRAF, FOXO1, KMT2D, CARD11, NOTCH1, CD79A, TP53, CDKN2B, ID3, MYC, CDKN2A, TCF3, EP300
Tosolini et al. (50)	NHL	CCL2, IL6ST, IDO1, TIMP1, LGALS3, VEGFA, HAVCR2, MRC1, TIGIT, CD163, IL10, PDCD1LG2, CTLA4, LAG3, LGALS1, CSF1, MSR1, JAK2, SOCS3, CD274, ICOS, HGF, IL23A, GDF15, FOXP3, PVR, MCL1, PDCD1, CCL22, LAIR1, CD86, IDO2, KIR2DL1
Mata et al. (33)	HL	EP300, BTK, CSF2RB, STAT6, CARD11, CSF1R, MYB, ABL1, B2M, BCL10, CD19, NFKBIA, CASP8, CD38, CREBBP, CSF2, FAS, LCP1, MYC, NOTCH1, PIK3CD, RET, SH3BP5, SMARCA4
de Jong et al. (32)^c^	DLBCL	ACTR2, ACTR3, ALOX5AP, ARPC2, ARPC3, ARPC4, ARPC5, BIRC3, BLK, BTK, CD19, CDK1, CNR2, CREB1, DCK, DHFR, ESR2, FDFT1, FNTA, GATM, GMDS, GPR18, HDAC1, HTR3A, ITPR1, LYN, MAP3K7, MAPK1, MDH1, METAP2, NPM1, PAPOLA, PARP1, PDK3, PLCG2, PPP1CA, PRKAB1, PRKD3, PSMD6, PSME3, QPCT, RAB8B, ROCK1, RPL19, RRM1, SDHC, SYK, UGCG, WEE1, XRCC4
Huet et al. (40)	FL	ABCB1, AFF3, ALDH2, CXCR4, DCAF12, E2F5, EML6, FCRL2, FOXO1, GADD45A, KIAA0040, METRNL, ORAI2, PRDM15, RASSF6, RGS10, SEMA4B, SHISA8, TAGAP, TCF4, USP44, VCL,VPREB1
Meng et al. (34)^d^	DLBCL HD	hsa-miR-127-5p, hsa-miR-136-5p, hsa-miR-154-5p, hsa-miR-3161, hsa-miR-337-3p, hsa-miR-34a-5p, hsa-miR-369-3p, hsa-miR-377-3p, hsa-miR-381-3p, hsa-miR-382-5p, hsa-miR-410-3p, hsa-miR-431-5p, hsa-miR-485-3p, hsa-miR-487a-3p, hsa-miR-494-3p, hsa-miR-496, hsa-miR-543, hsa-miR-654-3p, hsa-miR-656-3p, hsa-miR-889-3p
Xu et al. (51)	DLBCL	Pro-inflammatory genes: T effector (CD27, CD8A, GZMA, GZMB, IFN-G), IFN-G (CXCL10, CXCL9, IDO1, IFN-G, STAT1), APC (CD1C, CD40, TNFSF4). Anti-inflammatory genes: T regulatory (FOXP3), Th2 (IL10, IL13, IL4), Myeloid ((ARG1, IDO1, PTGS2), TCR signaling (CCL5, CD27, CD3D, CD3G, CD4, CD8A, IKZF3, IL2RB, PTPRCAP, TIGIT)
Liu et al. (44)	ENKTL	PPP2R2B, H2AFX, BRCA1, CCNA2, PKMYT1, TTK, MCM4, DNMT1, CHEK1, POLE2 PCNA, BRIP1, CDK2, IL2RB, E2F1, WEE, STMN1, CDC7, HIST1H3B, PTTG2, HIST1H3H, HIST1H3G, FANCB, EZH2, CDC6

*ALL, acute lymphoblastic leukemia; DLBCL, diffuse large B-cell lymphoma; HL, Hodgkin lymphoma; ENKTL, extranodal NK/T-cell lymphoma; FL, follicular lymphoma*.

a *Genes and SNPs (between parentheses) associated with resistance to both cisplatin and carboplatin*.

b *The signature was obtained in human lymphoma cell lines in vitro*.

c *Genes coding a protein for which a drug exists or is in development*.

d *The 20 miRNAs that regulate 21 key genes are listed*.

Asparaginase can induce severe toxic effects, as well as hypersensitivity reactions (HR) and liver toxicities. For these reasons, several studies have evaluated any possible association of these ADR with allelic variants, but findings were not consistent among trials. Indeed, in nearly 1,200 children affected by ALL, high throughput platforms find a relationship between HR and SNP rs4958351 belonging to the glutamate receptor *gria1* (76–78). Another study performed in several cohorts of ALL childhood patients (for a total of 3,308 children) found that the intronic SNP rs6021191 harbored by the nuclear factor of activated T cells 2 (*nfatc2*) gene displayed the strongest association with HR (57), whereas the *gria1* rs4958351 variant did not achieve the significance threshold of GWAS (*p* = 5 × 10^−8^). Finally, a recent study in 359 ALL children identified a highly significant correlation between the risk of developing HR and the HLA haplotype HLA-DRB1*07:01 / HLA-DQA1*02:01/HLADQB1*02:02 (59). Importantly, this study confirmed and expanded the results of the previous trial in ALL children that found a significant association of adverse reactions and the allele HLA-DRB1*07:01 (79).

Finally, it is possible to find significant correlations among the several ADRs of dexamethasone and few allelic variants in a gene. It is well-known that glucocorticoids have a plethora of toxic effects on several organs that may limit their use. Starting from a GWAS analysis, Ramsey and colleagues used an integrative analysis (PROMISE) to screen among several genetic variants that are mainly situated in regulatory regions (58). Interestingly, in 498 ALL children stratified according to variables as well as age, sex, race, and pharmacological treatments, the best candidate was the *f2RL1* gene that codes for a receptor involved in physiological processes, such as hemostasis, thrombosis and inflammation. In particular, two gene variants (rs2243057 and rs6453253) were highly significantly associated with osteonecrosis and thrombosis (58).

## New Platforms, Clinical Trials, and Companion Diagnostics

Clinical development of drugs takes certainly advantages from the introduction of these innovative platforms. European Medicines Agency (EMA) released guidelines regarding pharmacogenetic analyses during clinical trials, especially when the corresponding proteins play a role in drug pharmacokinetics (i.e., liver CYP isoforms) (80). In 2018, EMA prepared and made available the “*Guideline on good pharmacogenomic practice*” (81). The guideline enlists the principles deemed important to conduct genomic studies on germline DNA and to obtain high-quality findings that may influence treatment decisions, especially if they may explain interindividual variability in drug pharmacokinetics and pharmacodynamics (in terms of both efficacy and toxicity).

Three important questions should be considered: firstly, when newest platforms (i.e., microarrays, GWAS, NGS or ddPCR) should be employed; secondly, which type of study design is the most appropriate to exploit the pharmacogenetic information retrieved and finally how biomarkers could be transferred in clinical use as companion diagnostics.

With regards to the first question, it is clear that pharmacogenetic evaluations (by the most appropriate high-throughput platform) should be planned at the beginning of clinical evaluation of drugs, when the search for biomarkers may lead to important information regarding efficacy and tolerability. However, some issues are present both in phase I and II trials, because the limited number of patients required to identify and confirm the maximum tolerated dose in phase I and II studies could be a hurdle. These issues are probably amplified in early phase 1 studies (formerly phase 0 trials), which enroll few patients who receive doses lower than the starting dose of phase I trials. In these clinical studies, the search for biomarkers is mandatory, because the principal aim is to evaluate whether the drug may interact with its target. For example, the search of NGS or GWAS among hematological trials in lymphoma registered on the clinicaltrials.gov web database resulted in 33 phase I-III studies^1^. The early investigation of biomarkers could also result in a better allocation of resources (and time) in clinical trials. The majority of new drugs fail to achieve registration during phase III trials, which represent the most expensive phase in drug development. Therefore, biomarkers could early stop that development, and spare resources to expedite the evaluation of other molecules (1).

The second important issue is the rational planning of studies that include GWAS or NGS ancillary protocols. The retrospective genetic analysis of patients allows the identification of markers, which should be validated in independent cohorts. However, the available biomarker should guide the stratification of enrolled patients, so that a prospective phase is starting (82). At that time, targeted designs are chosen (83): marker carriers will receive the experimental drug, while the control arm will enroll marker-positive individuals (enrichment design) or biomarker-negative patients (biomarker strategy design) (84). These trial designs offer the advantage that the differences in clinical endpoints are amplified across study arms, even if this objective may not be always achieved (85). Master protocols, including the so-called basket and umbrella trials, may investigate several therapeutic options/alternatives in the same disease or *vice versa* (86).

The exploitation of the knowledge accumulated during preclinical experiments and first clinical trials may lead to the elaboration of companion diagnostics. In practical terms, these companion diagnostics may be laboratory tests that (a) have been developed during clinical trials in parallel with the drug, (b) allow the stratification of patients according to the presence/absence of the drug target, its transcriptional level or mutational status and (c) identify those patients who will benefit from drug administration with the highest probability (or who will experience the utmost severe toxicity). National and supranational regulatory bodies (i.e., EMA, Food and Drug Administration) have a major role in this area and they drive the search for these innovative instruments for personalized medicine, through assessment, performance^2^ and implementation. A practical exercise of how biomarkers (and companion diagnostics) could be useful for drug management is represented by registries of the Italian National Drug Agency (Agenzia Italiana del Farmaco, AIFA). For example, the prescription of antineoplastic drugs is mainly depending on the patients' status with respect to biomarkers, hence the evaluation of PD-L1 or cyclin D1 expression is mandatory for the prescription of nivolumab in cHL patients or ibrutinib in MCL individuals, respectively^3^. Furthermore, an interesting analysis performed by Ocana et al. clearly demonstrates that drugs with a companion diagnostic have an improved profile of tolerability, with special reference to gastrointestinal, cutaneous and neurological adverse events (87). Therefore, the parallel development of the drug and its companion diagnostics could facilitate market authorization and therapy management.

However, some issues may limit the complete and wide adoption of these approaches in clinical practice. For example, the number of targets within the genetic signature increases the detection of the responding genotype, but that subgroup of patients could represent a minority of the entire population (1) and it is a matter of debate (88). Moreover, adequate sample size, depth of sequencing and replicates are essential to perform robust NGS experiments (89). In order to overcome these hurdles, a joint working group from the Association of Molecular Pathologists and the College of American Pathologist prepared consensus recommendations about development, optimization, and validation of NGS (90), paying particular attention to the assessment of potential sources of error and their solutions through planned experiments, validation, and quality controls.

## Discussion and Conclusions

The millennial pharmacology and modern medicine are now dissecting molecular pathways of diseases and leading to an accelerated discovery of new pathological targets as it happens for the JAK/STAT, BRT, WNT, CTLA4-TIGIT, and PD-1/PDL1-2 pathways. Genetic variants and expression levels within these pathways may turn into predictive markers of activity/tolerability of newer drugs. The identification of actionable proteins means the possibility to synthesize targeted drugs and, when possible, to combine a companion diagnostic to expedite (and secure) the stratification of patients at diagnosis and over treatments. Interestingly, a recent survey in 9 countries (Argentina, China, France, Germany, Italy, Spain. and U.K.) involving 895 oncologists found that 90% of physicians have access to biomarker tests (91), suggesting that newest targeted therapies need molecular analyses.

However, these innovative approaches display some limits that may hinder their translation into effective tools for clinical routine (92). From reading the previous paragraphs, the reader can organize these issues into three categories. The first category refers to technical problems, including those related to poor quantity/quality of the biological material, method sensitivity, and its robustness and reliability: significant improvements have been introduced in techniques and many others will come soon. The adoption of new technical solutions (i.e., bisulfite staining or enrichment in NGS) adds new possibilities in the evaluation of pharmacogenetic targets. Even computer technology and bioinformatics contribute to surpass the problems related to the high quantity of collected data to find fit-for-purpose genetic signatures predictive of outcome and/or tolerability (93).

The second group of issues includes study planning, by which the advantage of a predictive signature (one marker or a panel) for a specific targeted therapy could be investigated appropriately. The retrospective design does not guarantee the availability of data and/or biological samples. Moreover, randomized controlled trials may fail to capture the advantage of target therapy for a biomarker-positive group of patients. Therefore, prospective master protocols and, in general, biomarker-based designs could offer a solution.

The third category collects those confounding factors that are invariably present in clinical trials: some of these variables (i.e., drug-drug interactions) are well-known, they can be detected, and their final effect on clinical outcome or tolerability can be weighted. On the contrary, other conditions are more complicated, as well as concomitant drugs (i.e., bone marrow growth factors) that may mask signs and symptoms of toxicity (neutropenia, anemia) associated with chemotherapy.

Finally, further criticisms may be enlisted. For example, 10% of physicians had not access to biomarkers tests for their patients (91) because the tests are too expensive or they are not locally available. Interestingly, physicians perceived that only 23% of patients were fully informed while some of them refused the tests (“*their patients did not want to delay treatment for testing or did not understand the benefits of testing*”). Moreover, patients may have further concerns about genetic testing, such as privacy, test affordability and lack of utility especially in older people or when therapies are effective (94). Indeed, patients' stratification based on the sole individual biological information does not represent the entire story, because patients' preferences and beliefs could be additional factors in precision/personalized medicine (95, 96).

In conclusion, the innovative platforms are playing a growing influence on precision medicine, from the search for predictive biomarkers, across a wide landscape of possible targets, up to their application in clinical routine as diagnostics. Some issues remain and are hampering the complete translation of findings into robust signatures, but many efforts are trying to implement these new approaches into clinical practice.

## Author Contributions

AD planned the manuscript and wrote it. EA, GL, and FC screened international scientific literature and prepared short summaries. SG and RD commented and revised the manuscript, while all of the authors accepted the final version.

### Conflict of Interest

The authors declare that the research was conducted in the absence of any commercial or financial relationships that could be construed as a potential conflict of interest.

## Footnotes

^1^Web site: www.clinicaltrials.gov (accessed, January 4th, 2019).

^2^European Medicine Agency - https://www.ema.europa.eu/documents/presentation/presentation-evolving-framework-co-development-medicinal-products-companion-diagnostics-falk-ehmann_en.pdf (accessed January 4th, 2019).

^3^Web site www.aifa.gov.it - Registries OPDIVO and IMBRUVICA (accessed, June 23rd, 2019).
